# Rare fungal infectious agents: a lurking enemy

**DOI:** 10.12688/f1000research.11124.1

**Published:** 2017-10-31

**Authors:** Anna Skiada, Ioannis Pavleas, Maria Drogari-Apiranthitou

**Affiliations:** 1 1st Department of Medicine, Laiko Hospital, School of Medicine, National and Kapodistrian University of Athens, Athens, Greece; 2 Intensive Care Unit, Laiko Hospital, Athens, Greece; 3 Infectious Diseases Research Laboratory, 4th Department of Internal Medicine, School of Medicine, National and Kapodistrian University of Athens, Athens, Greece

**Keywords:** invasive fungal infections, rare, yeast, mold, mucormycosis, trichosporon

## Abstract

In the expanding population of immunocompromised patients and those treated in intensive care units, rare fungal infectious agents have emerged as important pathogens, causing invasive infections associated with high morbidity and mortality. These infections may present either as *de novo* or as breakthrough invasive infections in high-risk patients with hematologic malignancies receiving prophylactic or empirical antifungal therapy or in patients with central venous catheters. Diagnosis and treatment are challenging. Physicians should have a high index of suspicion because early diagnosis is of paramount importance. Conventional diagnostic methods such as cultures and histopathology are still essential, but rapid and more specific molecular techniques for both detection and identification of the infecting pathogens are being developed and hopefully will lead to early targeted treatment. The management of invasive fungal infections is multimodal. Reversal of risk factors, if feasible, should be attempted. Surgical debridement is recommended in localized mold infections. The efficacy of various antifungal drugs is not uniform. Amphotericin B is active against most yeasts, except *Trichosporon*, as well as against *Mucorales*, *Fusarium*, and some species of *Paecilomyces* and dimorphic fungi. The use of voriconazole is suggested for the treatment of trichosporonosis and scedosporiosis. Combination treatment, though recommended as salvage therapy in some infections, is controversial in most cases. Despite the use of available antifungals, mortality remains high. The optimization of molecular-based techniques, with expansion of reference libraries and the possibility for direct detection of resistance mechanisms, is awaited with great interest in the near future. Further research is necessary, however, in order to find the best ways to confront and destroy these lurking enemies.

## Introduction

The quest for the early diagnosis and effective treatment of invasive fungal infections is ongoing, and there are still many obstacles to overcome. The main agents responsible for these infections are members of the genera *Candida*, *Cryptococcus*, and *Aspergillus*. However, there are other fungi, yeasts, and molds, which, though rare in human disease, can cause equally serious and life-threatening infections in immunocompromised and other susceptible hosts. These include yeasts ( *Trichosporon*, *Magnusiomyces*, *Saprochaete*, *Rhodotorula*, *Saccharomyces*, and *Malassezia*), mucormycetes, hyaline molds, dematiaceous (melanized) molds, and some novel taxa of thermally dimorphic fungi. Furthermore, a new *Candida* species, *C. auris*, has emerged and may pose a threat to hospitalized patients. The aim of this review is to present an update on invasive infections due to these rare and emerging fungi, focusing on new developments in diagnostic methodologies and therapeutic modules. In the light of recent taxonomic research and nomenclature changes of many pathogens, this review, though clinically oriented, will inevitably refer to some of these recent advances.

## Rare yeasts

Infections due to yeasts other than *Candida* and *Cryptococcus* account for about 2 to 3% of all fungemias ^1– 3^. They mainly occur in patients with hematologic malignancies or the critically ill. A large proportion of these infections represent breakthrough fungemias in patients already receiving an antifungal agent or are associated with central venous catheters (CVCs). Some of these yeasts are colonizers of the skin or the mucosal surfaces, whereas others may be found in the environment or even in food. The clinical presentation is non-specific, the only characteristic being that cutaneous exanthems are more common than in candidemia ^4^. Diagnosis is based on blood cultures.

### *Candida auris* 

*C. auris* is an emerging, multiple-drug-resistant (MDR) yeast causing a broad range of healthcare-associated invasive infections. It was first reported in Japan in 2009 when an isolate was recovered from the external ear canal of an inpatient ^4^. The first bloodstream infections were reported two years later from South Korea ^5^; shortly after that, *C. auris* fungemia was documented in India ^6– 8^, South Africa ^9^, and Kuwait ^10^. Since then, this difficult-to-treat yeast has been spreading across several countries in Europe, Asia, and the Americas ^11– 15^. In a recent study from India, *C. auris* accounted for more than 5% of candidemia in a national survey of intensive care units (ICUs) ^7^. Whole genome sequencing (WGS) and epidemiological analyses, performed by Lockhart *et al*., led to the conclusion that this species has emerged recently, independently, and almost simultaneously on three continents and not as a result of worldwide dissemination of a dominant clone ^12^. The earliest case to date was identified in retrospect by DNA sequencing of a Korean bloodstream isolate from 1996 ^5^.

Most cases of fungemia due to *C. auris* have been reported from hospitalized patients. Patients range from neonates to the elderly and have the well-known risk factors for invasive candidiasis. Like other *Candida* spp., *C. auris* colonizes the skin and mucosal surfaces (that is, genitourinary, gastrointestinal, and respiratory tract). In addition, environmental sampling of the clinical area surrounding colonized patients demonstrated contamination of the floor around bed sites, radiators, equipment monitors, mattresses, beds, windowsills, chairs, infusion pumps, and countertops ^16, 17^.

The ** phylogeny of *C. auris* is similar to that of *C. haemulonii*, which is another species resistant to amphotericin B and fluconazole ^5^. WGS data also revealed a relationship to *C. lusitaniae*, which is intrinsically resistant to many antifungals ^18, 19^. Commercial identification systems, such as Vitek 2 and API20C-AUX, misidentify this yeast as *C. haemulonii*, *C. famata*, *C. sake*, *Saccharomyces cerevisiae*, and *Rhodotorula glutinis* and this misidentification is primarily due to a lack of *C. auris* in the database of these systems ^5, 6, 9^. The most reliable methods for diagnosis of *C. auris* are molecular-based ones, such as amplified fragment length polymorphism (AFLP) fingerprinting and sequencing analysis ^20, 21^. Matrix-assisted laser desorption/ionization time-of-flight mass spectrometry (MALDI-TOF MS) systems (Bruker and Vitek MALDI-TOF MS) also have the potential for an accurate identification given the incorporation of a library containing *C. auris* and a proper extraction method ^21^. In a recently published study, where the isolates from 54 patients were analyzed, 93% were found to be resistant to fluconazole, 35% to amphotericin B, and 7% to echinocandins, while 41% were resistant to two antifungal classes and 4% were resistant to three classes ^12^. Voriconazole minimum inhibitory concentrations (MICs) were elevated in 50% of isolates in two large series from India and the Centers for Disease Control and Prevention (CDC) ^12, 20^. Posaconazole and isavuconazole show excellent *in vitro* activity against *C. auris* ^9, 12, 14^. Currently, the drugs of choice for treating fungemia due to *C. auris* are the echinocandins provided that susceptibility testing has been performed ^22^. A new drug, SCY-078, which is an orally bioavailable 1,3-β-D-glucan synthesis inhibitor, exhibits potent activity against *C. auris* isolates and may prove to be an important antifungal in the treatment of MDR species ^23^.

The overall crude in-hospital mortality rate of *C. auris* fungemia ranges from 30 to 60% ^16, 24^. The rapid emergence of *C. auris* has prompted the CDC in the USA, the European Centre for Disease Prevention and Control, and other institutions to publish alerts and recommendations regarding the control of this notorious fungus ^25, 26^.

### *Trichosporon* 

*Trichosporon* species are basidiomycetous yeast-like fungi, are found in soil and freshwater, are part of the human mycobiome, and colonize the skin, the peri-genital areas, and the gastrointestinal tract. Invasive trichosporonosis has been described in patients with leukemia, burns, and AIDS, in patients receiving corticosteroids, and in patients who have undergone heart valve or ophthalmic surgery ^27– 31^. *Trichosporon* spp. cause a wide spectrum of infections, ranging from white piedra and hypersensitivity pneumonia in immunocompetent patients to fungemia, endocarditis, peritonitis, and meningitis in patients with hematologic malignancies ^27, 31– 34^. The species most commonly found in fungemias are *T. asahii* (75%), *T. mucoides* (7%), *T. inkin* (5%), and *T. asteroides* (5%) ^28^, but at least 15 other species have been identified from clinical samples ^29^. Microscopic characteristics are pseudohyphae and septate hyphae along with arthroconidia and blastoconidia.

Diagnosis is made by blood cultures. Species identification relies either on phenotypic methods or on molecular analysis targeting the ribosomal DNA internal transcribed spacer (ITS) region ^35^. The rapid MALDI-TOF MS methodology is being increasingly applied and appears to be a promising tool. Molecular methods have also been developed for the detection of *Trichosporon* spp. in blood and tissues, even in formalin-fixed and paraffin-embedded (FFPE) tissue ^36^, but no standardized method exists yet. Cross-reactions with the serum *Cryptococcus* glucuronoxylomannan antigen as well as the *Aspergillus* galactomannan antigen (GM) have been reported in several cases of *Trichosporon* fungemia ^37– 39^, and dual positivity may be suggestive for *Trichosporon* infection. Serum β-D-glucan is often detected, but sensitivity is low ^39^.

The preferred antifungal for treating trichosporonosis is voriconazole ^40^. Posaconazole and other triazoles are also active, and fluconazole shows variable potency ^40– 42^. Amphotericin B, the echinocandins, and flucytosine are not indicated for treating trichosporonosis. The mortality of disseminated infection remains high (rates of 42 to 77% ^27, 32, 43^).

### *Magnusiomyces* and *Saprochaete* 

*Magnusiomyces capitatus* (formerly *Geotrichum capitatum*, *Blastoschizomyces capitatus*, or *Saprochaete capitata*) and *Saprochaete clavata* (formerly known as *Geotrichum clavatum*) are human pathogens that are closely related and are frequently mistaken for each other ^44^. The taxonomy of *Geotrichum* was revised relatively recently ^45^, but many authors use the older names. With the use of ITS sequencing, isolates are identified as *S. clavata*, *M. capitatus*, or *Geotrichum candidum* ^44^. These fungi are ascomycetous yeasts found in soil, water, plants, poultry feces, and dairy products ^46– 48^. They may colonize the human skin and mucosae and can cause infections clinically similar to candidiasis. Reports of infections due to *M. capitatus* originate mainly from Europe, mostly from the Mediterranean area ^43, 49, 50^, but there have also been publications from Asia ^51^. The majority of cases are seen in patients with underlying hematologic malignancies ^43, 49, 52^. Nosocomial outbreaks have been reported ^49, 53^. Fungemias due to *S. clavata* are very rare, but a multicenter outbreak was identified by WGS in France ^54^. In patients with neutropenia, the disease is disseminated, often presenting with fever and cutaneous lesions. Several organs, including the lung, liver, spleen, bone, kidneys, brain, and endocardium, may be involved ^50^. Keratitis ^55^, meningitis, and osteomyelitis ^56^ may also occur.

Diagnosis is based on blood cultures. Molecular methods are necessary in order make a correct identification ^44^. Patients with invasive infection may have a positive GM test, but it is uncertain whether this can be used as a diagnostic tool because invasive aspergillosis may coexist with infection due to *Magnusiomyces* or *Saprochaete* ^57, 58^.

The preferred agent for the treatment of invasive infections is amphotericin B with or without concomitant flucytosine ^40^. Voriconazole is active *in vitro*, and there are case reports where it has been used successfully ^46, 59^. Mortality of disseminated infection is high, ranging from 50 to 75% ^43, 47, 60^.

### *Rhodotorula* 

*Rhodotorula* spp *.* are basidiomycetous yeasts, which are widespread in nature. They are found in air, soil, lakes, seawater, foods, and beverages ^61^. They have also been isolated from medical equipment, such as bronchoscopes, and from shower curtains, bathtubs, and toothbrushes ^62^. The fungus may colonize the skin, nails, and the respiratory, gastrointestinal, and genital tract of humans ^63^.

The culture of *Rhodotorula* spp. produces distinctive orange to salmon-colored mucoid colonies. The most common species, which may cause invasive infections in humans, are *R. mucilaginosa (rubra)*, *R. glutinis*, and *R. minuta*. Infections occur mainly in patients with hematologic or other malignancies and in the majority present as fungemia, which is almost always catheter-related ^63– 68^. Other reported infections include endocarditis ^64, 69, 70^, peritonitis (associated with ambulatory peritoneal dialysis) ^71^, meningitis ^72^, and endophthalmitis ^67, 73^ and occur in patients with AIDS, extensive burns, or cirrhosis, in those who have undergone intra-abdominal surgery, and in critically ill patients in the ICU ^74, 75^.

*Rhodotorula* spp. are resistant *in vitro* to echinocandins and azoles ^76, 77^, and there are several cases where fungemia with *Rhodotorula* emerged in patients receiving prophylaxis with these agents ^66, 78^. The recommended treatment is amphotericin B with or without flucytosine ^40^. CVCs should be removed. Mortality is estimated to be 12 to 20% ^63, 64^.

### *Saccharomyces* 

*Saccharomyces cerevisiae* (also known as “baker’s yeast”) is a ubiquitous ascomycetous yeast found in soil, plants, and fruit ^79^. *S. boulardii* is a strain of *S. cerevisiae* with some specific genome features ^80, 81^ and is found in some probiotic preparations ^82, 83^ used in the prevention and treatment of various diarrheal diseases ^84^. *Saccharomyces* has been isolated from the throat, stool, urine, and perineum of patients with hematologic malignancies ^85^. It is an emerging cause of invasive fungal infections, either in patients receiving probiotics ^82, 83, 86, 87^ or in those with underlying diseases, such as leukemia ^79^. There have been reports of nosocomial outbreaks ^88, 89^, some of them attributed to central catheters or hand transmission ^88, 90^, but the largest proportion of these fungemias is associated with probiotics ^79^. The clinical presentation is the same as that of candidemia. Diagnosis is made by culture and conventional identification methods ^40^. In the search for early diagnostic markers, β-D-glucan has been considered and has been found in the plasma of patients and in culture supernatant ^91, 92^, but no clinical data are available to prove its usefulness. A novel multiplex real-time polymerase chain reaction (PCR) assay for the detection and differentiation of fungal pathogens in clinical specimens of hematological patients, including *Saccharomyces* spp., has been developed and seems promising, but validation is still required ^93^. Amphotericin B has the lowest MICs for *Saccharomyces* and is considered the drug of choice for its treatment ^40^. There have been reports of successful treatment with echinocandins ^94, 95^. Fluconazole is often inactive. Probiotics should be discontinued and CVCs removed ^40^. Mortality of *S. cerevisiae* fungemia was 29.5% in one study but, in most cases, could not be attributed only to fungemia ^87^.

### *Malassezia* 

*Malassezia* is a genus of basidiomycetous yeasts comprising the lipid-dependent *Malassezia furfur* and the lipid-independent species *Malassezia pachydermatis*, which are part of the normal skin microbiota ^96^. They can cause skin infections (for example, pityriasis versicolor) as well as systemic infections in neonates receiving lipid-containing parenteral nutrition and in children and adults with hematologic malignancies, cancer, Crohn’s disease, and chronic ambulatory peritoneal dialysis ^97^. There are multiple reports of outbreaks in neonatal ICUs ^98– 100^. The clinical signs and symptoms of *Malassezia* fungemia are non-specific, consisting mainly of fever. Dissemination to the heart, lungs, and other organs may occur ^101^. Diagnosis is made by culture but is difficult because routinely used media do not support the growth of this fungus, which requires lipids ^101^. The recommended treatment for severe cases is amphotericin B and for non-severe cases fluconazole, although *M. pachydermatis* may be less susceptible *in vitro* ^40, 97, 101^. The mortality of invasive *Malassezia* infections is unknown, but it seems that, with appropriate management, the attributable mortality is low ^101^.

## Rare molds

### Mucormycetes

Mucormycosis is the third most common cause of invasive fungal infection (after candidiasis and aspergillosis). The agents of mucormycosis are ubiquitous in nature and are found mainly in decaying organic matter. They belong to the order *Mucorales* and include the genera *Rhizopus*, *Rhizomucor*, *Mucor*, *Lichtheimia*, *Apophysomyces*, *Cunninghamella*, *Saksenaea*, *Cokeromyces*, *Actinomucor*, and *Syncephalastrum* ^102^. Some of these genera, such as *Saksenaea* and *Apophysomyces*, are more common in certain geographic areas ^103– 105^. The major risk factors for mucormycosis are uncontrolled diabetes mellitus, hematologic malignancies, organ or bone marrow transplantation, neutropenia, treatment with corticosteroids, trauma and burns, and deferoxamine (an iron/aluminium chelator) therapy ^106^. Iron acquisition is a critical step in the pathogenetic mechanism of mucormycosis, and deferoxamine has been shown to act as a siderophore for *Mucorales* ^106^.

*Mucorales* cause a diverse and devastating spectrum of infections. Rhinocerebral, pulmonary soft tissue, and disseminated disease are the most common clinical presentations, but virtually any organ can be affected ^107, 108^. The fungi invade the arteries, leading to extensive vessel thrombosis and subsequent infarction ^106^. Although tissue necrosis is the hallmark of mucormycosis, a presentation and syndrome-oriented approach to diagnosis lacks sensitivity and specificity. Other fungi, such as *Aspergillus* or *Fusarium*, may produce the same clinical signs. Nevertheless, there are some features which should lead to a higher index of suspicion for invasive pulmonary mucormycosis ^109^. These include prior prophylaxis with voriconazole or emergence of breakthrough fungal infection in an immunocompromised patient receiving agents active against *Aspergillus* but not *Mucorales*. Radiologically, multiple (at least 10) nodules and pleural effusion are more common in mucormycosis. The presence of reverse halo sign on computed tomography is a strong indicator of lung mucormycosis and may appear earlier than other radiologic findings ^110– 112^.

The diagnosis is traditionally based on direct microscopic examination, histopathology, and culture. *Mucorales* produce rapidly growing white to gray fluffy colonies with broad hyphae microscopically and a variety of species-specific structures such as stolons, rhizoids, sporangia, apophyses, sporangiophores, columellae, and sporangiospores. The hyphae are large, ribbon-like, irregular pauciseptate or aseptate in contrast with those seen in aspergillosis, which are more regular and septate. However, mucormycosis can be either overdiagnosed or missed by histopathology. In a study of 58 culture-proven mold infections from four tertiary centers using a blinded assessment, *Aspergillus* spp. infections were misidentified as mucormycosis in 11% of the cases ^113^. In another study, 2 out of 58 cases diagnosed as aspergillosis on histopathology or cytology or both were proven to be mucormycosis by culture ^114^. Furthermore, 30 to 50% of histopathology-proven cases may have negative cultures ^115, 116^.

Studies have shown that molecular identification of mucormycosis is accurate, using the ITS region of the ribosomal DNA as a first-line sequencing target for the identification of Zygomycete organisms in pure culture ^117^. When no culture is available, PCR assays on fresh or FFPE tissues can identify and discriminate between agents of aspergillosis and mucormycosis ^118– 123^. These techniques are not yet standardized but provide a promising tool for the identification of fungal agents. Detection of circulating *Mucorales* DNA in serum using real-time PCR is another field of current research and may aid in the earlier diagnosis of the disease ^124, 125^.

Early diagnosis of mucormycosis is crucial, and prompt therapeutic intervention may prevent progressive tissue invasion and its sequelae ^109, 126^. Therefore, it is very important to use all available diagnostic methods in order to achieve the best possible outcome. Treatment is multimodal, including antifungal agents, surgical debridement, and reversal of underlying risk factors ^127, 128^. *Mucorales* are resistant to most antifungals *in vitro*. Amphotericin B is the most active drug, except for *Cunninghamella* and *Apophysomyces* isolates ^129, 130^. Posaconazole and isavuconazole are also active, while itraconazole and terbinafine show some activity against certain strains. There seems to be some correlation between the degree of susceptibility of *Mucorales* isolates to amphotericin B and outcomes. In a small study by Lamoth *et al*., an MIC of not more than 0.5 μg/mL was significantly associated with better 6-week outcomes ^131^. Liposomal amphotericin B is the drug of choice for first-line treatment. Posaconazole is recommended for salvage or maintenance therapy. Isavuconazole has recently been approved for the treatment of mucormycosis, but its role as first-line treatment has not yet been defined ^132^.

Even with aggressive therapy, the mortality of mucormycosis remains high. For this reason, there is interest in the use of combination treatment or adjunctive modalities. In a recent study, which evaluated the impact of monotherapy versus combination therapy in a group of 106 patients with hematologic malignancies and which used a propensity score analysis, there was no improved outcome for the group receiving combination treatment ^133^. Combination of amphotericin B with deferasirox, an iron chelator which does not act as a siderophore for *Mucorales*, was associated with poorer outcomes in a small prospective study in patients with hematologic malignancies ^134^. However, based on preclinical data, there might be a role for this approach in patients with other underlying diseases ^135^. Other adjunctive treatments used in the attempt to enhance the outcome of this devastating disease include cytokines and hyperbaric oxygen. Some authors published case reports of successive treatment of mucormycosis with the use of hyperbaric oxygen in combination with antifungal treatment ^136^. There are also some data showing that granulocyte-macrophage colony-stimulating factors or interferon-gamma or both may enhance the immune response against certain *Mucorales* ^137^. However, since the data are only *in vitro* or pre-clinical, these therapies must be used cautiously.

### Hyaline molds

***Fusarium*.** *Fusarium* species are widely distributed in nature, occurring in ecosystems all around the globe. They are known important plant pathogens ^138^ and ** constitute a large genus with a complex and evolving taxonomy. However, only a few species complexes (SCs), currently 10, are involved in human disease; the most frequent are *Fusarium solani*, *F. oxysporum*, *F. incarnatum-equiseti*, *F. fujikuroi* (including the species *F. verticillioides*, *F. proliferatum*, and *F. sacchari*), *F. clamydosporum*, and *F. dimerum* ^139, 140^. Depending on the immune status of the host and the portal of entry, they cause a broad spectrum of infections, including superficial, locally invasive, and disseminated infection, and have considerable morbidity and high mortality rates ^141^. Some species produce mycotoxins and have a significant impact on animal and human health ^138^.

In immunocompetent hosts, *Fusarium* species, along with *Candida* and *Aspergillus*, are considered a leading cause of keratitis. Risk factors include accidental trauma, long-term use of topical corticosteroids and antibiotics, diabetes, pre-existing eye infections, eye surgery ^142, 143^, and (since the 1980s) contact lens use, with increasing frequency ^144^, especially in more developed countries ^145– 148^. *Fusarium* keratitis progresses slowly and can cause devastating ocular damage after the formation of penetrating ulcer and endophthalmitis. Therapy consists of topical antifungals, sometimes in combination with subconjunctival injections, but therapeutic keratoplasty may be needed in difficult cases. In many cases, the outcome is poor, especially in delayed diagnosis, and is more favorable in contact lens wearers ^149^.

Other forms of fusariosis, including the most prevalent ones in otherwise healthy individuals, are onychomycosis and skin infections, peritonitis following continuous ambulatory peritoneal dialysis, catheter-associated fungemia, and, less frequently, pneumonia, osteomyelitis, arthritis, otitis, sinusitis, and brain abscess, usually after trauma ^150^.

In the immunocompromised hosts, those mostly at risk have acute myeloid and acute lymphoid leukemia, and the most important risk factors are neutropenia, lymphopenia, graft-versus-host disease, corticosteroid use, or any other immunosuppressive treatment. Infection is almost always invasive and frequently disseminated ^151^. The most common routes of infection are direct inoculation and airborne uptake ^141^, and the portals of entry are the paranasal sinuses, the lungs, and skin lesions such as interdigital intertrigo, abnormal appearing nails, and paronychia ^152, 153^. Disseminated fusariosis is hardly distinguishable from aspergillosis both clinically and histologically, but agents of fusariosis, unlike those of aspergillosis and mucormycosis, can be isolated from blood cultures. It typically presents with fever in severely neutropenic patients and with myalgia and sudden appearance of erythematous, papular, or painful nodular skin lesions which evolve rapidly to central necrosis. The lungs and sinuses can also be affected, and pneumonia occurs in almost 50% of cases ^154^.

According to the few existing epidemiological studies, the incidence appears to be low—less than 0.3% among allogeneic hematopoietic cell transplantation (HCT) recipients and patients with hematologic malignancies—but variable in different geographic regions ^155– 158^. The highest incidence is in Brazil: 5.2% among allogeneic HCT recipients and 3.8% in patients with acute myeloid leukemia ^158^. Hematological patients in this region appeared to have an unusually high incidence of cutaneous portal of entry ^159, 160^. In previous studies, increased incidence of infection has been associated with hospital water distribution systems which also serve as a potential reservoir of *Fusarium* spp. ^3, 161^. The distribution of *Fusarium* species varies too in different geographic regions, but it seems that any species of *Fusarium* can cause any type of infection ^162, 163^.

Diagnosis is based on conventional mycological methods—direct microscopy, culture, and histopathology ^154^—and has to be as prompt and accurate as possible. However, direct microscopy and histopathology, though highly sensitive, are not specific for *Fusarium*, as hyphae share the same characteristics with all hyaline molds, such as *Aspergillus* and *Scedosporium*.

Culture has low sensitivity, as it can be positive in just 60% of cases ^154^. Identification of a *Fusarium* isolate to the genus level is easy because of the characteristic banana-shaped macroconidia ^154^. However, identification to the species level requires molecular methods, preferably performed in reference laboratories ^140^. MALDI-TOF MS appears to be a promising and more feasible approach for the identification of clinical isolates of *Fusarium* at the species level provided that the existing database is expanded ^164^. For direct and rapid detection in tissues and blood, multiplex reverse transcription–PCR methods have recently been developed with promising results ^140, 165^.

Clinically important species are resistant to almost all currently used antifungals, and this poses a challenge for antifungal therapy ^166^. The exact resistance mechanisms in *Fusarium* have not been elucidated yet, but combinations of CYP51A amino-acid alterations or CYP51A gene overexpression or both might be involved in azole resistance ^167^. Amphotericin B has the lowest MICs *in vitro*, whereas the azoles show variable values with a large interspecies variability ^163^. Voriconazole appears to be effective *in vivo* and a good alternative to amphotericin B despite higher MICs ^154^.

Based on the available data, voriconazole or a lipid-based amphotericin B formulation is recommended as primary therapy ^151, 154^. Combination therapy is frequently used but not scientifically supported. Posaconazole is recommended as salvage therapy ^151, 154^. A thorough clinical evaluation and treatment of skin lesions, particularly onychomycosis, have been recommended for patients at high risk for invasive fusariosis, prior to or upon initiation of antineoplastic therapy ^154^. In addition to antifungal treatment, surgical debridement of infected tissues, removal of venous catheters in confirmed catheter-related fusariosis, and reversal of the immunocompromised state should take place whenever possible for an optimal outcome ^151, 154^. Prognosis in the immunocompromised is poor and is highly dependent on the underlying condition and immune status of the patient, but survival seems to have substantially increased in the last decade ^168^.

***Scedosporium and Lomentospora*.** The hyaline molds known as *Scedosporium* and *Pseudallescheria* until 2014 are cosmopolitan saprophytes, commonly found in soil, polluted water, compost, and the manure of cattle and fowl ^151, 169^. They have a complex taxonomy, hence the nomenclature discrepancies with older studies ^170– 172^. The most frequent clinically relevant species are *Scedosporium boydii*, *S. apiospermum*, and *S. auratiacum*. *S. boydii* and *S. apiospermum* are clearly distinct species but, owing to common pathology characteristics and antifungal susceptibility patterns, may be considered a complex and indicated as “ *S. apiospermum* SC” in the routine laboratory, as proposed by de Hoog *et al*. in 2013 ^173^. The name “ *Scedosporium*” has also been proposed for their teleomorphic stages (sexual forms) *Pseudallescheria boydii* and *P. apiosperma* ^172^.

Distribution varies according to climate ^151^. They cause highly lethal opportunistic infections in immunocompromised patients, but also a wide range of infections in immunocompetent individuals, with considerable morbidity ^171^. They are frequently isolated from patients with cystic fibrosis, occasionally causing lung infection ^174, 175^.

Infections in the immunocompetent occur after penetrating trauma and include keratitis, endophthalmitis, otitis, sinusitis, central nervous system (CNS) infections, osteoarticular and soft tissue infections, and mycetoma ^176, 177^. Disseminated infections with *S. apiospermum/Pseudallescheria* and pneumonia or CNS involvement can follow near-drowning accidents in polluted water ^171, 177– 185^. Of note are cases of transplant recipients with previously near-drowning victims as donors ^186, 187^. In contrast, *Lomentospora prolificans* (formerly *Scedosporium prolificans*, or *S. inflatum*) is mainly isolated from cases with underlying immunosupression ^188^. Infection can affect any organ, usually the skin, sinuses, lungs, and the CNS, following hematogenous dissemination, and delayed treatment of brain abscesses is associated with a high mortality rate (>75%) ^188^. This species is also phylogenetically separated from the other *Scedosporium* species; therefore, the recommendation was recently made to rename it *Lomentospora prolificans* ^172^ (that is, under its oldest genus name *Lomentospora* ^189^).

As in fusariosis and other mold mycoses, diagnosis is based on direct microscopy, culture, and histopathology, but more sensitive and specific methods are sought for accurate and prompt diagnosis. New molecular methods have recently been developed and are promising for both direct detection and identification to the species level. In infections with *L. prolificans*, blood cultures may be positive in more than 50% of cases and isolates can be easily differentiated from their *S. apiospermum* counterparts because of their characteristic microscopic morphology with inflated phialides ^171^.

Therapy is challenging because of a high rate of resistance to antifungals, and the therapeutic outcome is usually poor in patients with persistent immunosuppression. *S. apiospermum* is resistant *in vitro* to amphotericin B and flucytosine, and susceptibility to itraconazole, voriconazole, posaconazole, and the echinocandins is variable. *L. prolificans* is highly resistant to all antifungals, and mortality rates are as high as 95% in immunocompromised patients ^190^. Voriconazole demonstrates the strongest *in vitro* activity against this species ^191^. There are suggestions of synergistic activity of voriconazole with colistin ^192^, terbinafine ^193– 197^, or caspofungin ^198^. Combinations of azoles and terbinafine and sequential azole and terbinafine therapy also appear effective against *L. prolificans* ^199^. In general, the management of both *S. apiospermum* SC and *L. prolificans* depends on the underlying condition of the host, and voriconazole has been suggested as first-line treatment ^200– 204^. In the immunocompromised, therapy is multimodal, and, whenever possible, reversal of the immune suppression, along with surgical debridement of infected tissues, is recommended ^205, 206^.

***Paecilomyces and Purpureocillium*.** Until recently, the fungal species *Paecilomyces variotii* and *Paecilomyces lilacinus* were considered to belong to the same genus, but new taxonomic studies have shown that they are not genetically related, and the latter is now assigned the name *Purpureocillium lilacinum* ^207^. They are found in soil, decaying plant material ^208– 210^, indoor air, and foods ^210^.

*Paecilomyces* infections are associated with almost any organ or system of the human body ^211^. The most usual infections in immunocompromised patients are cutaneous, catheter-related, and disseminated infections, pneumonia, cellulitis, fungemia, and pyelonephritis ^208, 209, 211– 213^. The most frequent infection caused by *Purpureocillium lilacinum* is keratitis in otherwise healthy hosts after trauma, surgery, or prolonged contact lens use ^214^.

Diagnosis is based on conventional mycological methods. MALDI-TOF MS analysis is a promising tool, as with all of the above-described fungi, but requires further cutoff standardization and a reliable reference library ^215^. There is no optimal antifungal treatment established. *P. lilacinum* strains are highly resistant to amphotericin B but susceptible to azoles, whereas *P. variotii* is usually amphotericin B susceptible ^151^.

### Dematiaceous (or melanized) molds

These fungi are darkly pigmented because of the presence of melanin in the cell wall, which acts as a virulence factor ^216– 218^. More than 70 genera are implicated in human disease; *Alternaria*, *Aureobasidium*, *Bipolaris*, *Cladophialophora*, *Curvularia*, *Exophiala*, *Exserohilum*, *Hortaea werneckii*, *Neoscytalidium dimidiatum*, *Ochroconis*, and *Rhinocladiella* are some of the most known representatives ^219^.

Commonly found in soil and plant material, they are distributed worldwide, although some neurotropic fungi are often geographically restricted: *Rhinocladiella mackenziei* occur in the Middle East and *Cladophialophora bantiana* occur mainly in India, whereas cases of *Exophiala dermatitidis* are reported mainly from East Asia ^220^. They cause phaeohyphomycosis, a term that refers to a broad range of diseases ranging from allergic sinusitis to superficial and disseminated infections, pneumonia, brain abscess ^220^, and even iatrogenic infections, such as the 2012–2013 multistate (USA) fungal meningitis epidemic due to *Exserohilum rostratum* ^221^. Infections can be life-threatening in both immunocompromised and immunocompetent individuals. Diagnosis is based on conventional mycological methods and PCR. There are no standardized therapies, but voriconazole, posaconazole, and itraconazole demonstrate the most consistent *in vitro* activity against this group of fungi. Voriconazole may be superior for CNS infections. Surgery is required for local infections and brain abscesses ^220^.

## Rare and emerging dimorphic fungi

### *Emmonsia-*like fungi and *Emergomyces* 

The genus *Emmonsia* makes up part of the *Ajellomycetaceae* family of the *Onygenales* order, along with the genera *Blastomyces*, *Histoplasma*, and *Paracoccidioides*, among others. Until recently, two species were recognized— *E. crescens* and *E. parva*—which cause adiaspiromycosis, a granulomatous pulmonary disease of rodents and other small mammals and very rarely of humans, through conversion into large, thick-walled “adiaspores” in the infection sites ^222, 223^. However, in recent years, there has been an increase in cases of diseases caused by novel *Emmonsia*-like species. The increase between 2006 and 2015 was primarily driven by the recognition of HIV-associated cases in South Africa following the introduction of molecular identification protocols for dimorphic fungal infections in 2008 ^223^. These new species differ from the “classical” ones ( *E. crescens* and *E. parva*) in that they are able to convert into yeast-like cells capable of replication and extra-pulmonary dissemination during the course of infection, instead of the non-replicating adiaspores ^223^. Infection probably occurs through the inhalation of spores, but the environmental niche is as yet unknown. New taxonomic research suggests some putative virulence factors that may play a role in the infection and pathogenicity of the novel *Emmonsia* strains, but these findings need further investigation ^224^. The species *Emmonsia pasteuriana* ^225– 227^ has now been classified in a new genus of the *Ajellomycetaceae* family, *Emergomyces* ( *Emergomyces pasteurianus* comb. nov.) ^228^.

Clinical diagnosis of infections due to *Emmonsia*-like species is difficult because of the protean nature of symptoms and signs. Infections usually are disseminated involving primarily the skin and lungs and frequently are misdiagnosed as tuberculosis. Cutaneous manifestations may be misdiagnosed as varicella, Kaposi’s sarcoma, and drug reactions ^229^. Laboratory diagnosis is made by biopsy of the skin or any other involved organ and blood culture, although histopathologic findings are not sufficient to distinguish *Emmonsia* or *Emergomyces* sp. from other dimorphic fungi. In culture, the above genera are hard to identify by morphological methods alone, and molecular identification is imperative for accurate diagnosis. Urine *Histoplasma* antigen may aid diagnosis because of high cross-reactivity ^229^. *Emmonsia*-like infections are highly lethal if untreated, the most effective drug being amphotericin B ^229^, although some azoles appear to have variably low MICs *in vitro* ^222^.

*Es. pasteurianus* and other *Emergomyces* species ( *Es. africanus* and *Es. orientalis*) isolated from sporadic cases in different countries such as Italy, Spain, China, Canada, South Africa, and Germany underline the potential of *Emergomyces* species as new cosmopolitan opportunistic pathogens in the immunocompromised host ^228^.

### *Talaromyces (Penicillium) marneffei* 

*Talaromyces marneffei* (formerly *Penicillium marneffei*), recently renamed on the basis of new sequence data ^230^, has been recognized as a significant human pathogen across Southeast Asia with the global AIDS pandemic ^231^ and represents an “AIDS-defining pathogen” in this region ^232, 233^. However, in recent years, improved treatment of HIV infection with highly active antiretroviral therapy and control of HIV/AIDS because of improvement in the healthcare systems of developing countries in that region, such as mainland China, Thailand, and Vietnam, have led to a change in the epidemiology of *T. marneffei* infection, and there is an increasing number in immunocompromised non-HIV-infected patients ^234^. These are usually patients receiving immunosuppressive therapies associated with transplantation or autoimmune diseases and hematology patients treated with novel targeted therapies, including anti-CD20 monoclonal antibodies and kinase inhibitors ^234– 236^, but also patients with primary adult-onset immunodeficiency due to anti-interferon-gamma auto-antibodies ^237^. A donor-related transmission in a lung transplant recipient was also recently reported ^238^. Bamboo rats are considered the only identified natural reservoir so far, and transmission is believed to occur through conidia inhalation ^234, 235^. As patients with penicilliosis may present all over the world following global travelling, a good travel history is essential for clinical diagnosis ^239^.

The symptoms include fever, weight loss, anemia, lymphadenopathy, hepatosplenomegaly, respiratory signs, and often molluscum contagiosum-like skin lesions mostly on the face and neck, described as translucent papules, umbilicated with a central necrotic depression ^235, 240^. However, clinical diagnosis is challenging, especially in the absence of skin lesions and in non-HIV patients.

Laboratory diagnosis is based on histopathology, direct microscopy, and cultures. Blood cultures are frequently positive, and bone marrow cultures are positive in nearly all cases ^241^. The morphological identification is based on the characteristic colony of the mold phase of the fungus at 25°C with red pigment in the agar and yeast-like arthroconidia in the Gram stain of cultures at 37°C. This finding is crucial, as *T. marneffei* is the only dimorphic fungus in the *Talaromyces* genus. New techniques with much shorter turn-around times, such as real-time PCR and MALDI-TOF, are being developed with promising results ^242, 243^, but, as with the other rare fungi, increased sensitivity and an expanded database are required in order for them to be introduced into routine practice.

The treatment of choice is intravenous amphotericin B (liposomal formulation 3 to 5 mg/kg per day or lipid complex formulation 5 mg/kg per day) for 2 weeks, followed by oral itraconazole (400 mg/day) for 10 weeks; alternatively, voriconazole can be used (6 mg/kg twice a day on day 1, followed by 4 mg/kg twice a day for at least 12 weeks) ^244, 245^.

## Conclusions

Rare fungal infectious agents have emerged as important pathogens in recent years because of the expanding population of immunocompromised patients. These fungi are ubiquitous saprobes or plant pathogens and lurk to invade any vulnerable host. The infections they cause are associated with high morbidity and mortality, whereas diagnosis and treatment are challenging. Early diagnosis is of paramount importance but requires a high index of clinical suspicion and accurate diagnostic methods. Conventional diagnostic methods such as direct microscopy, cultures, and histopathology are still essential, but rapid and more specific molecular techniques are needed for early targeted treatment. The currently used PCR-based methods and others, such as MALDI-TOF MS, appear promising but need optimization.

The management of invasive fungal infections is multimodal, and, whenever feasible, surgical debridement and reversal of risk factors are recommended. The efficacy of various antifungals is not uniform, and many of these infectious agents are intrinsically resistant to multiple drugs; in addition, recommendations are mainly based on clinical case experience. Combination treatment, though recommended as salvage therapy in some infections, is controversial in most cases. Despite the use of available antifungals, mortality remains high.
